# Supplementary material for: Comprehensive Characterization of a Novel Bacteriophage, vB_VhaS_MAG7 against a Fish Pathogenic Strain of Vibrio harveyi and Its In Vivo Efficacy in Phage Therapy Trials
Source: Int J Mol Sci. 2023 May 3;24(9):8200. doi: 10.3390/ijms24098200 (PMC10179652; doi:10.3390/ijms24098200)

Figure S1. VIRIDIC generated heatmap incorporating intergenomic similarity values (right half) and alignment indicators (left half and top annotation).

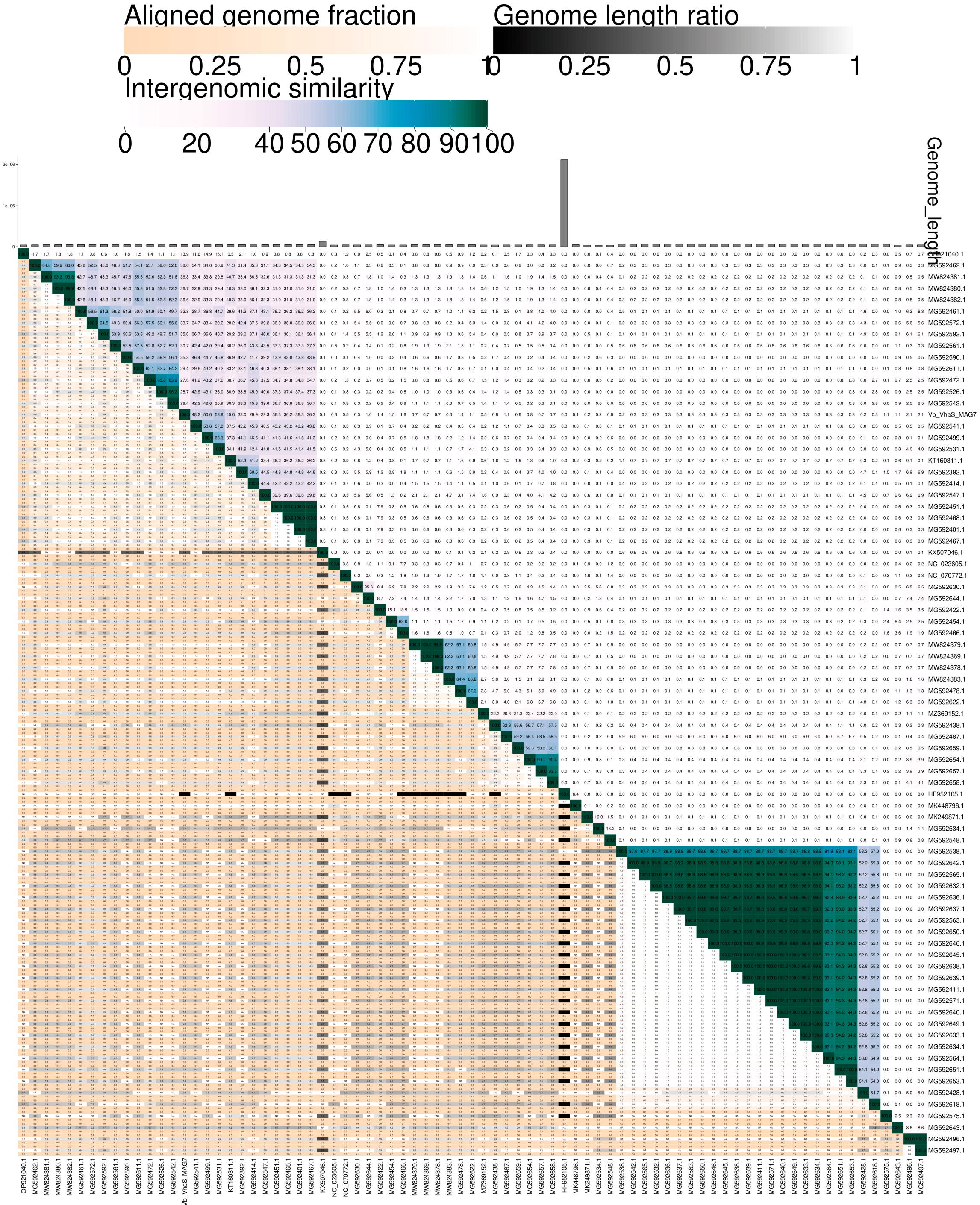

Supplement: Supplementary file 1 [file ijms-24-08200-s001.zip › ijms-2240000-supplementary.pdf]
